# Differential Inhibitory Mechanisms of Myricetin and Dihydromyricetin on α-Glucosidase: A Combined Molecular Docking, Isothermal Titration Calorimetry and Surface Plasmon Resonance Study

**DOI:** 10.3390/foods15152707

**Published:** 2026-07-31

**Authors:** Zhaoqi Jiang, Yuhan Wang, Litao Jiang, Rui Zhang, Xiaoyang He, Meng Meng, Anjun Liu, Min Zhang, Jiaping Zhou

**Affiliations:** 1 State Key Laboratory of Food Nutrition and Safety, Tianjin University of Science & Technology, Tianjin 300457, China; 2 College of Food Science and Engineering, Tianjin University of Science & Technology, Tianjin 300457, China; 3 State Key Laboratory of Chinese Medicine Modernization, Tianjin University of Traditional Chinese Medicine, Tianjin 301617, China; 4 Haihe Laboratory of Modern Chinese Medicine, Tianjin University of Traditional Chinese Medicine, Tianjin 301617, China; 5 Research Centre of Modern Analytical Technology, Tianjin University of Science & Technology, Tianjin 300457, China

**Keywords:** α-glucosidase inhibitors, flavonoids, molecular docking, isothermal titration calorimetry (ITC), surface plasmon resonance (SPR)

## Abstract

α-Glucosidase inhibitors (AGIs) significantly regulate blood glucose by delaying carbohydrate digestion and slowing glucose absorption, thus playing a critical role in glycemic control. Structurally, dihydromyricetin (Unless otherwise stated, the term dihydromyricetin used throughout this manuscript refers to trans-(2R,3R)-(+)-dihydromyricetin.) differs from myricetin in that the C2=C3 double bond in the C-ring is saturated, resulting in a dihydroflavonol instead of a flavonol. This study investigated the inhibition mechanism of α-glucosidase by the C2=C3 double bond structure using a set of integrated and multi-perspective approaches combining enzyme kinetics, multi-spectroscopic methods, molecular docking, isothermal titration calorimetry (ITC), and surface plasmon resonance (SPR). Myricetin (IC_50_ = 13.648 ± 0.157 μM) was found to be a more potent α-glucosidase inhibitor than dihydromyricetin (IC_50_ = 453.922 ± 1.643 μM). Enzyme kinetics indicated that myricetin acted as a competitive inhibitor, whereas dihydromyricetin functioned as a non-competitive inhibitor. To further examine these interactions, multi-spectroscopic analysis demonstrated that binding of myricetin caused significant changes in the microenvironment around fluorescent amino acids (such as tyrosine and tryptophan) in α-glucosidase, resulting in slight unfolding of the enzyme structure. Additionally, molecular docking provided a detailed molecular perspective, identifying hydrogen bonding and hydrophobic interactions as the primary forces driving the binding of two flavonoids to α-glucosidase. Delving deeper into the binding mechanism, ITC analysis provided thermodynamic evidence that myricetin (K_D_ = 6.215 ± 0.022 μM) exhibited a stronger binding affinity to α-glucosidase than dihydromyricetin (K_D_ = 232.648 ± 1.236 μM), with both interactions being enthalpy-driven and primarily mediated by hydrogen bonds. Building on this, SPR analysis offered additional insights into the binding process, showing that myricetin not only had a higher binding affinity (K_D_ = 3.416 ± 0.015 μM) but also a faster association rate (k_a_ = 1668 ± 23 M^−1^ s^−1^) compared to dihydromyricetin (K_D_ = 11.539 ± 0.056 μM, k_a_ = 339.7 ± 17.1 M^−1^ s^−1^). In conclusion, this study demonstrated that the C2=C3 double bond plays a key role in enhancing α-glucosidase/inhibitor interactions, providing a theoretical basis for the design of novel AGIs and proposing a new set of multi-perspective methods for elucidating these inhibition mechanisms.

## 1. Introduction

α-Glucosidase (EC 3.2.1.20) is an enzyme widely distributed in nature, playing a pivotal role in the metabolism of carbohydrates within living organisms [1,2]. This class of enzymes is present in a multitude of biological entities, including humans, animals, plants, fungi, and microorganisms [3,4,5]. In the human body, they primarily function on the brush border membrane of the small intestine [6]. Within the digestive system, α-glucosidase is the key enzyme in the final stage of carbohydrate digestion [7]. It is primarily responsible for catalyzing the hydrolysis of α-1,4 and α-1,6 glycosidic bonds, breaking down polysaccharides (such as starch, glycogen) and disaccharides (such as maltose, sucrose) into individual glucose molecules [8]. The released monosaccharides can be absorbed by the small intestine, leading to an increase in blood glucose levels. α-Glucosidase Absorption of the released monosaccharides in the small intestine elevates blood glucose levels. α-Glucosidase plays a key role in maintaining energy balance and glucose homeostasis and has been recognized as a primary target enzyme for the prevention and treatment of diabetes [9]. However, the lack of experimentally resolved three-dimensional structures for some mammalian α-glucosidases has limited the understanding of their molecular mechanisms. Homology models constructed based on isomaltase structures provide useful structural information for molecular docking and interaction analysis; however, differences in sequence identity and local conformational features may introduce uncertainties, and further validation by molecular dynamics simulations is required to assess model stability and reliability [10]. α-Glucosidase inhibitors (AGIs) can reduce the rate of carbohydrate digestion by restricting the breakdown of dietary carbohydrates, their absorption, and subsequent glucose production through inhibiting the degradation of linear or branched oligosaccharide units such as maltose, maltotriose, and limit dextrin. This prevents glucose absorption into the bloodstream and helps control postprandial blood glucose levels. Most AGIs display structural resemblance to disaccharides or oligosaccharides, enabling them to bind to the carbohydrate-binding site of α-glucosidase. These complexes exhibit greater binding affinity than the carbohydrate-glycosidase complex, competitively inhibiting various α-glucosidases located in the small intestine. Additionally, certain AGIs suppress carbohydrate hydrolysis in a non-competitive manner through the formation of a ternary complex with the glycosidase and its carbohydrate substrate [11]. The AGIs widely used in clinical practice include acarbose, miglitol, voglibose, and 1-deoxynojirimycin (1-DNJ), all of which possess a pseudo-oligosaccharide structure, competitively bind to the α-glucosidase active site, inhibit postprandial hyperglycemia by preventing glucose release from oligosaccharide hydrolysis, and increase insulin sensitivity while reducing the secretory burden on β-cells [12,13]. However, these chemical drugs are often associated with gastrointestinal discomfort, bloating, and diarrhea, in addition to being relatively costly [14]. Therefore, exploring novel AGIs derived from natural sources as potential alternatives holds significant research value.

Flavonoids, with their diverse biological activities and relatively low potential side effects, have emerged in recent years as promising candidates for the development of novel AGIs. These compounds are secondary metabolites found in plants and fungi, characterized by a 15-carbon skeleton consisting of two benzene rings (rings A and B) and a heterocyclic ring (ring C). Based on their structural features and varying degrees of oxidation, flavonoids are generally classified into six main categories: flavones, flavonols, flavanones, flavan-3-ols, anthocyanins, and isoflavones. Myricetin and dihydromyricetin are both flavonol compounds [15]. Flavonols, a subclass of flavonoids, are a group of natural phenolic compounds widely present in plants [16]. Their structural feature is the presence of a carbonyl group (-C=O) at the C-4 position of the C-ring, often accompanied by hydroxyl groups at various positions on the A- and B-rings [17]. Flavonols are extensively distributed in nature and are commonly found in foods such as fruits, vegetables, tea, and red wine [16]. Due to their diverse biological activities, flavonols have significant research value and application potential in the fields of food science, nutrition, and pharmacology. Myricetin and dihydromyricetin have similar basic chemical structures but also key differences. Myricetin contains a C2=C3 double bond, which is a conjugated double bond system extending from C2 to C3, forming part of the flavonoid compounds. The presence of the double bond endows myricetin with specific stereochemical characteristics that may affect its binding to biological targets [18]. The C2=C3 double bond also enhances its antioxidant capacity. Studies have shown that myricetin possesses antioxidant, anti-inflammatory, anticancer, antidiabetic, cardiovascular protective, neuroprotective, as well as antimicrobial and antiviral activities [19]. Dihydromyricetin, on the other hand, has the double bond at the corresponding C2-C3 position saturated; that is, an additional hydrogen atom is added, making this position a single bond [20]. The structural differences between the two may lead to differences in their biological activities and mechanisms of action. Studies have indicated that flavonols as α-glucosidase inhibitors show potential hypoglycemic effects [21]. Despite preliminary research affirming the potential of myricetin and dihydromyricetin as α-glucosidase inhibitors, comprehensive comparative studies on myricetin and dihydromyricetin are sparse, and the underlying mechanisms of their inhibitory effects remain largely unexplored [19]. The specific structural elements contributing to their differential inhibition have yet to be delineated through comparative investigation. Clarification of their inhibitory mechanisms and binding processes with the α-glucosidase could expedite the development of myricetin and dihydromyricetin as novel AGI drugs or dietary supplements [22]. These insights will significantly contribute to the design and optimization of new AGIs, leading to more effective inhibition of α-glucosidase activity, thereby slowing carbohydrate breakdown and helping to control blood glucose levels.

Some common endpoint methods, such as enzyme kinetics and fluorescence assays, have limitations in studying the inhibitory mechanisms of AGIs because the parameters obtained are mostly indirect and cannot directly provide information on the binding process of myricetin/dihydromyricetin with α-glucosidase. Therefore, more new methods need to be explored for investigating the inhibitory mechanisms of AGIs against α-glucosidase. Isothermal titration calorimetry (ITC) and surface plasmon resonance (SPR) are two widely used techniques in the study of biomolecular interactions and offer distinct advantages for investigating AGIs [23]. ITC, a label-free technique, provides quantitative information such as the binding constant (K_D_) and number of binding sites (n) [24]. Additionally, ITC enables real-time detection of subtle heat changes during the binding of myricetin or dihydromyricetin to α-glucosidase [25], allowing for accurate measurement of enthalpy (ΔH) and entropy (ΔS) changes in the binding process, thus yielding the thermodynamic parameters of the reaction [26]. Meanwhile, the binding process of myricetin/dihydromyricetin with α-glucosidase can be dynamically monitored in real-time and in a label-free manner using SPR technology [27]. When α-glucosidase binds to myricetin or dihydromyricetin, it results in changes in the local refractive index, thereby altering the SPR angle [28,29,30,31].

This study investigated the potential of natural flavonols as AGIs. Utilizing a combination of molecular docking, surface plasmon resonance (SPR), and isothermal titration calorimetry (ITC), it systematically evaluated the inhibitory mechanisms of myricetin and dihydromyricetin against α-glucosidase. The innovation lies in elucidating the critical role of the C=C double bond not only through the thermodynamic and kinetic analysis of molecular interactions, but also through AI-driven molecular docking. Collectively, these approaches revealed the inhibition mechanism from complementary angles of molecular simulation, molecular dynamics, and thermodynamics. This work provides theoretical insights for the design of key functional groups in flavonol-based AGIs and introduces novel methodologies for their development.

## 2. Materials and Methods

### 2.1. Chemicals and Materials

**Figure 1:**
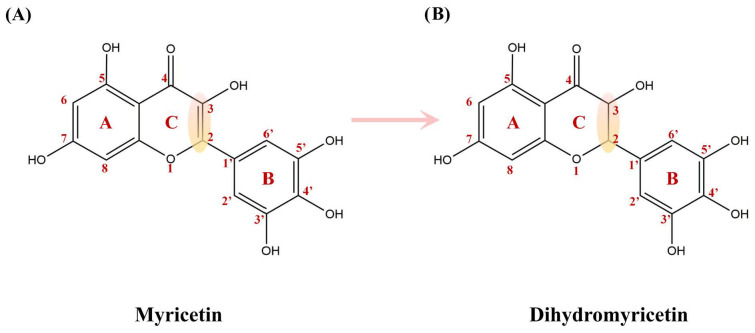
Chemical structure of two flavonoids. (**A**) Myricetin and (**B**) dihydromyricetin. The flavonoid skeleton consists of three rings (A, B, and C rings). The numbers indicate the standard atom numbering system of flavonoids, where unprimed numbers (1–8) represent atoms in the A and C rings, and primed numbers (1′–6′) indicate atoms in the B ring.

α-Glucosidase from Saccharomyces cerevisiae (EC 3.2.1.20, cat: G 0660, 750 units) was purchased from Sigma-Aldrich Co., Ltd. (St. Louis, MO, USA). Myricetin (purity ≥ 98%, HPLC; CAS No. 529-44-2) and trans-(2R,3R)-(+)-dihydromyricetin (purity ≥ 98%, HPLC; CAS No. 27200-12-0 were purchased from Shanghai Yuanye Bio-Technology Co., Ltd. (Shanghai, China). 4-nitrophenyl-α-D-glucopyranoside (p-NPG) was obtained from Shanghai Yuanye Bio-Technology Co., Ltd. (Shanghai, China). Throughout the entire experiment, 20 mM phosphate buffer (pH 6.8) was used, except for the SPR experiments which utilized the PBS-P+ buffer from Cytiva Corporation (Boston, MA, USA). The chemical structures are shown in Figure 1. Other chemical reagents were of analytical grade.

### 2.2. *α*-Glucosidase Inhibition Assay of Two Flavonoids

The inhibitory effects of myricetin and dihydromyricetin on α-glucosidase were evaluated utilizing an established assay system designed for enzyme inhibitor screening, which incorporates p-nitrophenyl-α-D-glucopyranoside (p-NPG) as the substrate. The enzymatic cleavage of the α-glucosidic bond within p-NPG by α-glucosidase results in the generation of 4-nitrophenol, a compound characterized by its absorbance peak at 405 nm [32,33]. Enzyme solution (0.5 U mL^−1^, 40 μL) and flavonoid solution (10 μL) were combined in a 96-well plate and pre-incubated for 5 min at 37 °C. The reaction was initiated by adding 50 μL of 3 mM p-NPG and allowed to proceed for 30 min at 37 °C. After addition of 100 μL of 0.1 M Na_2_CO_3_, the amount of released 4-nitrophenol was determined from the absorbance at 405 nm. Inhibition was calculated using Equation (1). Acarbose was used as a positive control and tested under the same experimental conditions as myricetin and dihydromyricetin. Its IC_50_ value was calculated using the same nonlinear regression model. The absorbance at 405 nm was measured using a microplate reader. The inhibition percentage (%) was calculated according to Equation (1):

(1) $$\mathrm{Inhibition}\;\mathrm{rate}\;\left(\%\right)\;=\;\frac{\left({\mathrm{OD}}_{\mathrm{control}}\;-\;{\mathrm{OD}}_{\mathrm{control}\;\mathrm{blank}})\;-\;({\mathrm{OD}}_{\mathrm{sample}}\;-\;{\mathrm{OD}}_{\mathrm{sample}\;\mathrm{blank}}\right)}{{\mathrm{OD}}_{\mathrm{control}}\;-\;{\mathrm{OD}}_{\mathrm{control}\;\mathrm{blank}}}\text{×}100\%$$

OD_sample_ and OD_control_ denote the absorbance values of the inhibitor-containing reaction and the enzyme–substrate control, respectively. OD_sample_ _blank_ and OD_control blank_ are the corresponding background readings measured without enzymatic product formation.

Concentration–response data were fitted in SPSS by nonlinear regression using the logistic model shown in Equation (2), from which IC_50_ values were obtained.

(2) $$Y=D+\frac{\left(U-D\right)}{1+{\left(\frac{X}{C_{50}}\right)}^{S}}$$

Y is the percentage inhibition, D and U are the lower and upper asymptotes, C_50_ is the inhibitor concentration producing 50% inhibition, and S describes the slope of the fitted curve.

### 2.3. Inhibitory Kinetics of Two Flavonoids on α-Glucosidase Activity

The inhibitory effects of myricetin and dihydromyricetin on α-glucosidase were evaluated using a method similar to the standard assay for α-glucosidase activity. This involved fixing the p-NPG concentration at 3 mM and adjusting the α-glucosidase concentration across 0.4, 0.6, 0.8, and 1 U mL^−1^. The enzyme was then exposed to the flavonol inhibitors at concentrations ranging from 0 to 100 μM. The enzymatic activity was quantified by measuring the absorbance change, which reflects the reaction rate over one minute [34,35].

To identify the inhibition type of each flavonoid, enzyme-kinetic data obtained at multiple substrate and inhibitor concentrations were analyzed by nonlinear Michaelis–Menten regression and corresponding Lineweaver–Burk double-reciprocal plots [36,37,38]. For inhibition-mode analysis, the α-glucosidase concentration was maintained at 0.5 U mL^−1^, while the initial reaction rates were measured at p-NPG concentrations of 1.0, 2.0, 4.0, and 8.0 mM in the presence of selected fixed concentrations of myricetin or dihydromyricetin. Lineweaver–Burk plots were then generated by plotting 1/v against 1/[S], where v represents the initial reaction rate and [S] represents the p-NPG concentration.

(3) $$\mathrm{Competitive}\;\mathrm{inhibition}\;\frac{1}{v}=\frac{K_{m}}{V_{max}}\left(1+\frac{\left[I\right]}{K_{i}}\right)\times \frac{1}{\left[S\right]}+\frac{1}{V_{max}}$$

(4) $$\mathrm{Non}\text{-}\mathrm{competitive}\;\mathrm{inhibition}\;\frac{1}{v}=\frac{K_{m}}{V_{max}}\left(1+\frac{\left[I\right]}{K_{i}}\right)\times \frac{1}{\left[S\right]}+\frac{1}{V_{max}}\left(1+\frac{\left[I\right]}{K_{i}}\right)$$

(5) $$Slope=\frac{K_{m}}{V_{max}}\left(1+\frac{\left[I\right]}{K_{i}}\right)$$

(6) $$Y_{intercept}=\frac{1}{V_{max}}+\frac{\left[I\right]}{K_{si}V_{m}}$$

In these equations, v denotes the reaction rate, V_max_ represents the maximum reaction velocity, [S] and [I] refer to the substrate and inhibitor concentrations, respectively, and K_m_ is the Michaelis–Menten constant. K_i_ represents the dissociation constant for inhibitor binding to the enzyme. It was determined from plots of 1/v against [I] at several fixed substrate concentrations, with its value corresponding to the absolute value of the x-coordinate at which the fitted lines intersected.

### 2.4. Fluorescence Spectra Measurements

To explore the fluorescence quenching properties of two flavonoids on α-glucosidase, myricetin and dihydromyricetin were dissolved in 20 mM PBS (pH 6.8) to prepare solutions with concentrations ranging from 4 µM to 80 µM. Subsequently, α-glucosidase (4 μM) was mixed with an equal volume of the inhibitor solution. The resultant mixture was then incubated for 30 min at 37 °C under controlled conditions. This approach helped to study in detail how these two compounds affect the microenvironment of α-glucosidase.

The fluorescence spectrophotometer was utilized to capture the emission spectra for the aforementioned solutions. The spectral recording spanned a range from 290 to 450 nm, with the excitation wavelength set at 280 nm. The slit widths for both excitation and emission were configured at 2.5 nm, and the scanning rate was maintained at 240 nm/min. Additionally, the photomultiplier tube (PMT) voltage was adjusted to optimize the detection of the fluorescence signal [39]. The synchronous scan technique was implemented to examine the alterations in the molecular structure of α-glucosidase upon interaction with flavonoids. Two specific wavelength intervals were selected: one with a difference (Δλ) of 15 nm and another with Δλ of 60 nm between the excitation and emission wavelengths [40]. For synchronous fluorescence measurements, emission spectra were recorded from 200 to 350 nm, while all other instrumental settings were maintained as described above. In addition, three-dimensional fluorescence spectroscopy was used to evaluate the effects of myricetin and dihydromyricetin on the structure of α-glucosidase. The excitation and emission wavelength ranges were set at 200–350 nm and 200–500 nm, respectively, with a scanning rate of 12,000 nm min^−1^ [41]. To minimize fluorescence distortions caused by UV-induced reabsorption and inner-filter effects, the measured fluorescence intensities were corrected using the following equation:

(7) $$F_{C}=F_{m}e^{(A1+A2)/2}$$

In this equation, F_c_ denotes the corrected fluorescence intensity of α-glucosidase, whereas F_m_ is the experimentally measured fluorescence intensity. A1 and A2 represent the absorbance values of the flavonoids at the excitation and emission wavelengths, respectively.

### 2.5. Molecular Docking

Molecular docking serves as a valuable tool for elucidating drug–protein interactions. The interactions between two flavonoids and α-glucosidase were examined using AutoDock (version 1.5.7) [42]. Because the experimentally determined three-dimensional structure of α-glucosidase is currently unavailable, a homology model was constructed using the α-glucosidase sequence retrieved from the NCBI database. Isomaltase from Saccharomyces cerevisiae (PDB ID: 3A4A), which showed 72% sequence alignment with the target enzyme, was selected as the optimal structural template [43]. The three-dimensional structures of myricetin and dihydromyricetin were obtained from the PubChem database and subsequently optimized using Chem3D software (version 18.1.2). Prior to docking, both the receptor and the ligand structures were subjected to energy minimization utilizing the MM2 algorithm, with all water molecules removed to streamline the model. Subsequently, Gasteiger–Hückel charges were applied, and essential hydrogens were incorporated using the AutoDock Tools (version 1.5.7) to prepare the structures for interaction studies [44]. Molecular docking simulations were conducted with AutoDock vina, employing a grid system with a spacing of 0.325 Å, encompassing a box of dimensions 110 × 120 × 120, oriented with its center at coordinates X = 8.89, Y = 0.306, Z = 1.917 [26]. The ligands were permitted to explore the entire binding region globally to identify the conformation with the lowest possible energy state. The predicted protein–ligand binding poses and interaction patterns were subsequently visualized using PyMOL (version 4.6) [45].

### 2.6. Molecular Dynamics Simulation

Molecular dynamics (MD) simulations were performed to investigate the stability and interaction characteristics of the α-glucosidase–myricetin and α-glucosidase–dihydromyricetin complexes using the AMBER 20 software package [46]. The optimal docking conformations were selected as the initial structures for MD simulations. The α-glucosidase protein was parameterized using the protein.ff14SB force field, while myricetin and dihydromyricetin were prepared using the General AMBER Force Field (GAFF).

The complexes were solvated in a periodic box with the TIP3P water model, maintaining a minimum distance of 1 nm between the complex and the box boundary. After energy minimization and system equilibration, each complex was subjected to a 40 ns production simulation.

The simulation trajectories were analyzed by calculating the protein backbone root-mean-square deviation (RMSD) [47], protein–ligand hydrogen bonds, van der Waals interaction energy, electrostatic interaction energy, and binding free energy. Representative conformations during the simulation were extracted and visualized using molecular visualization software.

### 2.7. Isothermal Titration Calorimetry

Thermodynamic measurements of the interactions between myricetin/dihydromyricetin and α-glucosidasewere conducted using an ITC calorimeter (MICROCAL ITC200, Malvern, UK) [48]. Both α-glucosidase and the flavonoid solutions were prepared in the same PBS containing 5% DMSO to minimize heat effects arising from buffer or solvent mismatch. A volume of 300 μL of α-glucosidase solution (2.0 μM) was placed into the sample cell, and a total of approximately 60 μL of 50 μM myricetin/dihydromyricetin solution was titrated into it at 37 °C [26]. The individual injection volume was 4 μL, with an initial injection volume of 0.8 μL. The interval between injections was set at 180 s, with an initial interval set at 60 s, while the cell was stirred at a speed of 750 rpm. The reference power was 5 μcal/s. Subsequently, the experimental data obtained were fitted to a theoretical titration curve to calculate several thermodynamic parameters, including the enthalpy change (ΔH), entropy change (ΔS), binding constant (K), and the number of binding sites (n) [49]. The calculations were performed using MicroCal software (version 7.5).

### 2.8. Surface Plasmon Resonance

Surface plasmon resonance (SPR) measurements were performed using a Biacore T200 system (Cytiva, Boston, MA, USA) equipped with an HC200M polycarboxylate hydrogel sensor chip (Xantec). PBS-P containing 5% DMSO was used as the running buffer. All buffers and samples were filtered through 0.22 μm membranes and degassed before analysis [50]. α-Glucosidase was covalently immobilized on the sensor-chip surface through amine coupling. After optimization of the protein immobilization conditions, the chip was activated using a standard amine-coupling kit. A freshly prepared 1:1 (*v*/*v*) mixture of 0.4 M 1-(3-dimethylaminopropyl)-3-ethylcarbodiimide hydrochloride (EDC) and 0.1 M N-hydroxysuccinimide (NHS) was passed over both channel 1, which served as the reference channel, and channel 2 at a flow rate of 10 μL min^−1^ for 420 s to activate the carboxyl groups on the chip matrix. α-Glucosidase was then injected in pulses over the activated surface. Finally, the remaining active ester groups were blocked by injecting 1.0 M ethanolamine (pH 8.5) for 420 s.

α-Glucosidase was diluted according to the previously optimized preconcentration conditions. Before analysis, the flavonoids were dissolved in 10 mM PBS and passed through a 0.22 μm membrane filter. SPR measurements were carried out at 25 °C and a flow rate of 30 μL min^−1^, with association and dissociation phases of 120 s for each analyte concentration. Following each injection cycle, the sensor chip was regenerated using 10 mM glycine–HCl buffer (pH 2.0) at 30 μL min^−1^ for 30 s. The resulting binding sensorgrams were processed with the Biacore T200 Evaluation Software (version 3.2.1) using numerical integration and global fitting, and the kinetic data were analyzed with a simple 1:1 Langmuir interaction model [50]. Fitting of the flavonoid–α-glucosidase interaction data yielded the association rate constant (ka, M^−1^ s^−1^), dissociation rate constant (kd, s^−1^), and equilibrium dissociation constant (K_D_, μM), which reflects the binding affinity [51]. For sensorgrams that lacked clearly resolved association and dissociation phases, K_D_ was determined by steady-state affinity analysis. Before fitting, the response from the reference channel was subtracted from each sensorgram to minimize contributions from non-specific binding and bulk refractive-index changes.

### 2.9. Statistical Analysis

Data were reported as mean ± SE, and each experiment was performed independently in triplicate. The SPSS software package (version 18.0) was used in order to perform a statistical evaluation to determine significant differences. A *p*-value < 0.05 was considered statistically significant. A one-way analysis of variance (ANOVA) was performed on the data, followed by Tukey’s test of significant difference for multiple comparisons. All plots were performed using Origin 2019.

## 3. Results

### 3.1. Inhibition of *α*-Glucosidase by Two Flavonoids

The inhibitory activity of two flavonoids against α-glucosidase was dose-dependent. The half maximal inhibitory concentration (IC_50_) refers to the concentration of an AGI required to inhibit the activity of α-glucosidase by 50%. A lower IC_50_ value indicates a stronger inhibitory potency of the AGI against α-glucosidase, meaning that a smaller concentration of the compound is needed to achieve 50% inhibition. Myricetin (IC_50_ = 13.648 ± 0.157 μM) demonstrated significantly superior inhibitory effects compared to dihydromyricetin (IC_50_ = 453.922 ± 1.643 μM). Interestingly, despite the similar structures of myricetin and dihydromyricetin, there was more than a 30-fold difference in their inhibitory activities (*p* < 0.05), suggesting that the double bond on the C-ring of myricetin might be a key functional group for the inhibition of α-glucosidase [40]. Oxidation of C2=C3, which leads to structural changes and the formation of dihydromyricetin, may have reduced the activity of inhibiting α-glucosidase [52]. Under the same assay conditions, myricetin exhibited stronger α-glucosidase inhibitory activity than dihydromyricetin, with IC_50_ values of 13.648 ± 0.157 μM and 453.922 ± 1.643 μM, respectively. Compared with the positive control acarbose (IC_50_ = 0.338 ± 0.015 μM), both myricetin and dihydromyricetin showed weaker inhibitory activity, although myricetin displayed a relatively higher inhibitory potency among the two flavonoids.

### 3.2. Reversible Inhibition of α-Glucosidase by Two Flavonoids

**Figure 2:**
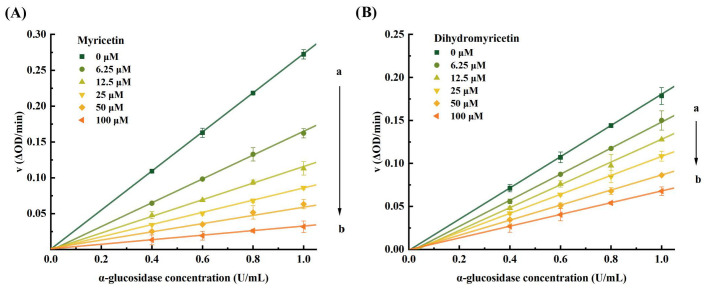
Reversible inhibition of α-glucosidase by two flavonoids. (**A**) Myricetin and (**B**) dihydromyricetin.

The inhibition mechanism of α-glucosidase activity by the two flavonoids was evaluated by plotting the reaction rate (v, expressed as ΔOD/min) versus α-glucosidase concentration (U mL^−1^) at different inhibitor concentrations. As shown in Figure 2, the lines representing the catalytic kinetics of α-glucosidase in the presence of myricetin or dihydromyricetin all passed through the origin, and the slope decreased with increasing inhibitor concentration. These results indicated that the inhibition of α-glucosidase by both flavonoids was reversible, with non-covalent interactions being present [53].

### 3.3. Kinetic Analysis of *α*-Glucosidase Inhibition by Two Flavonoids

**Figure 3:**
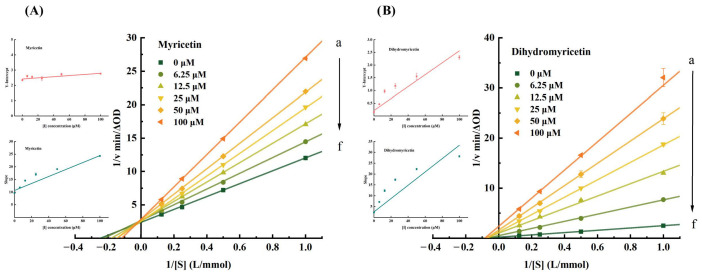
Lineweaver–Burk plots of the inhibitory effects of two flavonoids on α-glucosidase. (**A**) Myricetin and (**B**) dihydromyricetin.

In general, the classification of reversible inhibitory mechanisms encompasses competitive, non-competitive, uncompetitive, and mixed inhibition [54]. Accordingly, the inhibition modes of the two flavonoids were further assessed using Lineweaver–Burk double-reciprocal plots by examining the intersection patterns of the fitted lines in plots of 1/v against 1/[p-NPG] [37,55]. In a double reciprocal plot, the x-axis intercept of the line is the negative inverse of the Michaelis-Menten (−1/K_m_), and the y-axis intercept is the inverse of the maximum velocity (V_max)_ [56]. For myricetin, the fitted lines converged at the y-axis, indicating a competitive mode of α-glucosidase inhibition through the formation of an enzyme–inhibitor complex (Figure 3A) [40]. In contrast, the lines in the Lineweaver–Burk plot for dihydromyricetin intersected to the left of the y-axis in the negative x-coordinate region, indicating that dihydromyricetin inhibited α-glucosidase through a non-competitive mechanism (Figure 3B) [57]. The K_m_ values were unaffected, whereas the V_max_ diminished as the concentration of the compounds escalated [30]. Straight lines were obtained by plotting the inhibitor concentrations against the K_m_ values at various inhibitor concentrations. The K_i_ for myricetin and dihydromyricetin was determined to be (5.486 ± 0.043) μM and (140.612 ± 1.028) μM, respectively. The K_i_ represents the dissociation constant of the enzyme-inhibitor complex, with a lower K_i_ indicating a stronger binding affinity of the inhibitor to the enzyme or the enzyme-substrate complex [52]. Myricetin’s K_i_ value is lower than that of dihydromyricetin, indicating a stronger inhibitory effect of myricetin on α-glucosidase (*p* < 0.05) [34].

### 3.4. Endogenous Fluorescence Spectroscopy

**Figure 4:**
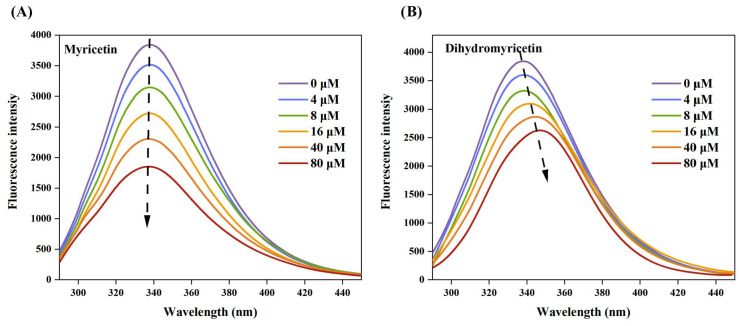
Endogenous fluorescence burst plots of two flavonoids against α-glucosidase. (**A**) Myricetin and (**B**) dihydromyricetin. c(α-glucosidase) = 4 μM, c(myricetin) = c(dihydromyricetin) = 4, 8, 16, 40, 80 μM. The black dashed arrows indicate the trend of λmax changes.

The interactions between α-glucosidase and the two flavonoids were investigated using multi-spectroscopic methods. The intrinsic fluorescence of many proteins primarily results from tyrosine (Tyr) and tryptophan (Trp) residues, which are highly sensitive to changes in their microenvironment. A decrease in intrinsic fluorescence intensity, defined as quenching, occurs when proteins interact with small molecules through static, collisional, or combined (static and dynamic) quenching mechanisms. Therefore, endogenous fluorescence spectroscopy was used to detect the binding of the two flavonoids to α-glucosidase. Figure 4 shows the fluorescence emission spectra of α-glucosidase recorded at an excitation wavelength of 280 nm. With increasing concentrations of myricetin or dihydromyricetin (ranging from 0 to 80 μM), there was a progressive attenuation of the fluorescence intensity of α-glucosidase, showing a dose-dependent effect. At the same time, myricetin exhibited a higher fluorescence quenching rate (51.75% ± 2.36%) compared to dihydromyricetin (31.51% ± 1.49%), signifying a more robust interaction between myricetin and α-glucosidase (*p* < 0.05), resulting in more pronounced structural alterations in the enzyme [39]. Additionally, dihydromyricetin was observed to induce a certain redshift (8.6 nm).

### 3.5. Synchronous Fluorescence Spectroscopy

**Figure 5:**
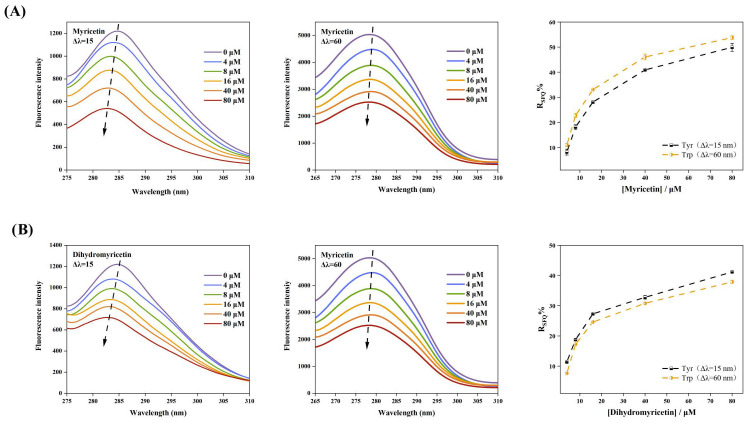
Simultaneous fluorescence burst plots of two flavonoids against α-glucosidase at measured wavelength differences (Δλ = 15 nm and 60 nm). (**A**) Myricetin and (**B**) dihydromyricetin. c(α-glucosidase) = 4 μM, c(myricetin) = c(dihydromyricetin) = 4, 8, 16, 40, 80 μM. The black dashed arrows indicate the the trend of λmax changes.

Synchronous fluorescence spectroscopy was employed to probe the local environments of tyrosine (Tyr) and tryptophan (Trp) residues and to assess associated conformational changes in the protein [58]. A Δλ of 15 nm was used to monitor fluorescence changes associated with Tyr residues, whereas a Δλ of 60 nm was selected to characterize the fluorescence response of Trp residues. Additionally, the polarity of the surrounding environment was known to influence the maximum emission wavelength (λ_max_) of the amino acid residues. Thus, alterations in the λ_max_ of the amino acid residues can be employed to ascertain the structural changes in α-glucosidase [40]. The synchronous fluorescence spectroscopy scanning results for the two flavonoids were depicted in Figure 5. As the concentration of myricetin and dihydromyricetin escalated, the synchronous fluorescence intensity of α-glucosidase progressively diminished, further corroborating the findings from the fluorescence quenching spectrum.

Because the fluorescence signals of both Tyr and Trp residues decreased significantly (*p* < 0.05), the synchronous fluorescence quenching ratio (RSFQ = 1−F/F0) was calculated to quantify the relative contributions of these residues to the overall fluorescence quenching of α-glucosidase [40]. In the presence of myricetin, Trp fluorescence was quenched more strongly than Tyr fluorescence, indicating a greater perturbation of the local environment around Trp residues and suggesting that the myricetin-binding region may be located closer to Trp. By contrast, dihydromyricetin produced more pronounced quenching of Tyr than Trp, implying that its interaction with α-glucosidase affected the microenvironment surrounding Tyr residues to a greater extent [59]. In addition, both myricetin and dihydromyricetin induced a slight blue shift in the maximum emission wavelength of Tyr residues in α-glucosidase, suggesting modest changes in the local microenvironment and conformation of the enzyme.

### 3.6. Three-Dimensional Fluorescence Spectroscopy

**Figure 6:**
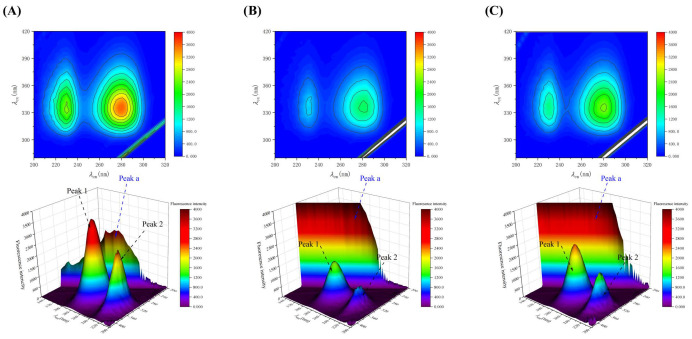
3D fluorescence burst plots of two flavonoids against α-glucosidase. (**A**) PBS + α-glucosidase, (**B**) myricetin + α-glucosidase, and (**C**) dihydromyricetin + α-glucosidase. c (α-glucosidase) = 4 μM, c (myricetin) = c (dihydromyricetin) = 80 μM.

Three-dimensional fluorescence spectroscopy was employed to study the conformational alterations of the protein that occurred when myricetin and dihydromyricetin interacted with α-glucosidase. To eliminate the influence of the inner filter effect, all fluorescence intensity data were corrected using the corresponding inner filter correction method before further analysis. As indicated in Figure 6, peak a represents the Rayleigh scattering peak (λ_ex_ = λ_em_). Peak 1 (λ_ex_ = 280 nm, λ_em_ = 340 nm), which mirrors the spectral actions of Trp and Tyr residues. Peak 2 (λ_ex_ = 230 nm, λ_em_ = 340 nm) mainly exposes the fluorescence stemming from the protein polypeptide backbone due to the n→π* transition [41]. A concentration of 80 μM myricetin and dihydromyricetin was added to the α-glucosidase solution, and the fluorescence intensity at peak 1 decreased to varying extents. This suggested that myricetin and dihydromyricetin interacted with Trp and Tyr, thereby suppressing fluorescence and increasing the hydrophobicity of the α-glucosidase microenvironment. Myricetin demonstrated a more pronounced reduction (43.20% ± 2.17%) than dihydromyricetin (26.93% ± 0.97%), signifying more substantial alterations to the microenvironments surrounding Trp and Tyr (*p* < 0.05). Furthermore, the introduction of myricetin and dihydromyricetin markedly lowered the fluorescence intensity of peak 2, suggesting a minor unfolding of the α-glucosidase protein structure. This outcome indicated that the secondary structure of α-glucosidase experienced modifications, findings that were in line with those from endogenous and synchronous fluorescence spectroscopy.

### 3.7. Molecular Docking Analysis

**Figure 7:**
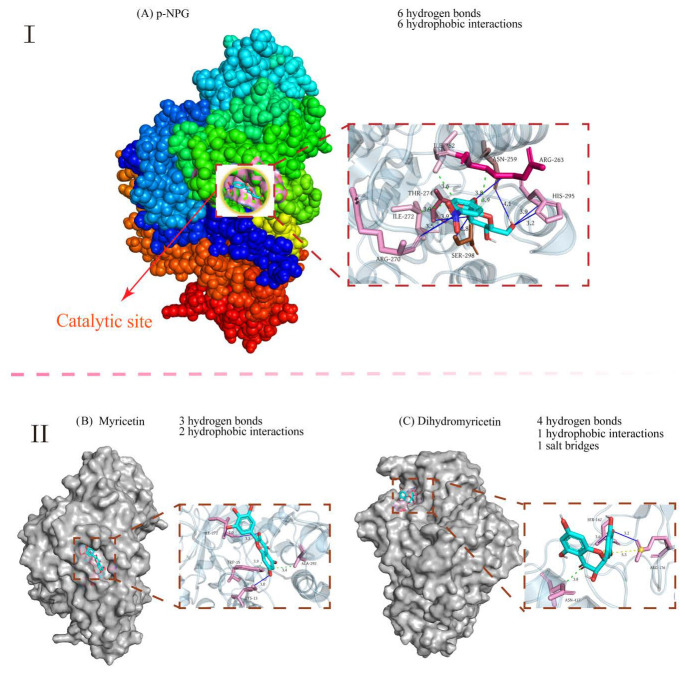
The molecular docking results. (**A**) p-NPG with α-glucosidase, (**B**) myricetin with α-glucosidase, and (**C**) dihydromyricetin with α-glucosidase. Blue straight line: hydrogen bonding, green dashed line: hydrophobic forces, orange line: π-stacking, yellow line: salt bridge.

Molecular docking studies, supported by PyMOL visualization, were performed to provide insights into the binding interactions between the two flavonoids and α-glucosidase at the molecular level [31]. Myricetin and dihydromyricetin ligands, together with p-NPG, were docked to the binding site of α-glucosidase [60]. As shown in Figure 7 and Table 1, myricetin was identified as a competitive inhibitor with a binding energy of −8.9 kcal/mol, which bound to the catalytic site of α-glucosidase, similar to p-NPG (−7.5 kcal/mol) [61]. The negative values of binding energy indicated that the interactions between the inhibitor and the α-glucosidase were exothermic, entailing the release of energy [30]. The greater the absolute value of the binding energy, the more stable the binding of the ligand to the protein. Therefore, myricetin, having a lower binding energy, binds more stably to α-glucosidase, directly preventing substrate binding [60]. It was observed that the -OH group at the 3’ position of the B ring and the -OH group at the 3 position of the C ring both interacted with the residue Ile272 through hydrogen bonds at distances of 2.47 and 2.12 Å, respectively. The double bond on the C-ring was likely to have significantly influenced the electronic distribution of myricetin, which may have enhanced its interactions with the catalytic site of α-glucosidase [62]. The A-ring of myricetin was noted to have extended into the cavity of α-glucosidase, where the 7-OH group interacted with the residue Lys13 through a hydrogen bond at a distance of 2.41 Å. At the same time, the C6 and C10 positions on the A-ring of myricetin formed two hydrophobic interactions with residues Ala292 and Trp15, respectively, at distances of 3.38 and 3.93 Å.

In contrast, dihydromyricetin exhibited weaker binding to the catalytic site of α-glucosidase, with a binding energy of −7.8 kcal/mol, much lower than that of myricetin. This explains why the inhibitory effect of myricetin on α-glucosidase is greater than that of dihydromyricetin. It was reported that the oxidation of the C-ring double bond might lead to changes in the molecular structure, potentially reducing the number of effective hydrogen bond donors or acceptors, and thereby decreasing the binding affinity of dihydromyricetin for the enzyme [63]. Additionally, it might also have reduced the conformational flexibility of the molecule, preventing it from binding to the enzyme’s competitive site as effectively as myricetin did. Therefore, this further confirmed that dihydromyricetin was a non-competitive inhibitor, consistent with the enzyme kinetics results [52]. Specifically, the 4′-OH group on the B-ring of dihydromyricetin formed two hydrogen bonds with the Arg176 residue of α-glucosidase at distances of 2.65 and 2.68 Å, and interacted with the Ser162 residue through a hydrogen bond at a distance of 3.17 Å. Upon comparison with myricetin, it was observed that dihydromyricetin exhibited elongated hydrogen bond lengths, which corresponded to diminished bond energies, consequently resulting in reduced binding affinities [64]. Simultaneously, two hydrophobic forces were observed to form at a distance of 3.56 Å between the C4 position on the C-ring of dihydromyricetin and the residue Asn417. Furthermore, a salt bridge was noted to form between the phenyl-ring of dihydromyricetin and the residue Arg176 at a distance of 5.21 Å [30,43]. This interaction was likely permitted by the spatial conformation of dihydromyricetin, which enabled it to approximate the charged residues on the α-glucosidase surface, thus facilitating salt bridge formation. The salt bridge is recognized as a robust non-covalent interaction that forms stable connections between a protein and its ligand [65]. This electrostatic interaction significantly increased the stability of the binding and reduced the dissociation of the ligand from the protein surface [66,67,68].

The outcomes of molecular docking studies suggested that hydrogen bonding and hydrophobic forces were likely the principal drivers of interaction for the two flavonol compounds with α-glucosidase. These results indicate that the binding between them was non-covalent in nature, and these docking findings corroborated the results from the kinetic investigations of enzyme inhibition. Moreover, the presence of the double bond on the C-ring was found to enhance the reactivity of myricetin, allowing it to engage in more potent hydrogen bonding with the catalytic site of α-glucosidase, leading to a more intimate association with the enzyme’s active site.

### 3.8. Molecular Dynamics Simulation Analysis

**Figure 8:**
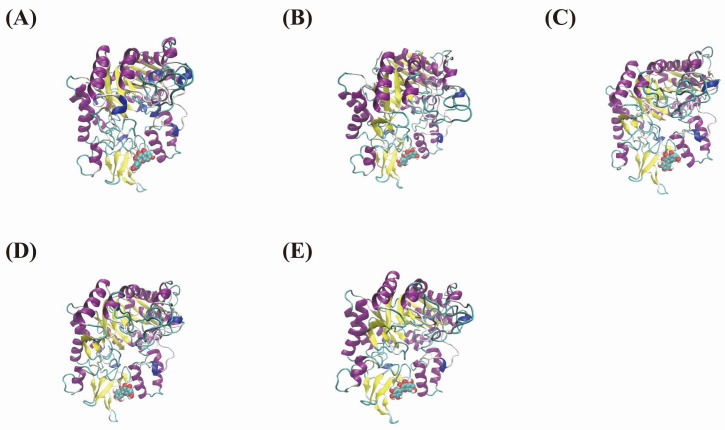
Representative conformations of the α-glucosidase–myricetin complex during the 40 ns molecular dynamics simulation. Structures were extracted at (**A**) 0 ns, (**B**) 10 ns, (**C**) 20 ns, (**D**) 30 ns, and (**E**) 40 ns. The protein is represented in cartoon mode, with different colors indicating different secondary structure elements, while the ligand molecules are shown as ball-and-stick models.

**Figure 9:**
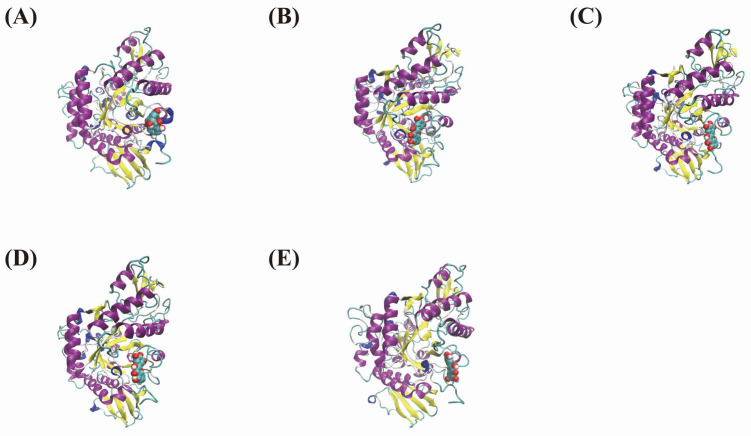
Representative conformations of the α-glucosidase–dihydromyricetin complex during the 40 ns molecular dynamics simulation. Structures were extracted at (**A**) 0 ns, (**B**) 10 ns, (**C**) 20 ns, (**D**) 30 ns, and (**E**) 40 ns. The protein is represented in cartoon mode, with different colors indicating different secondary structure elements, while the ligand molecules are shown as ball-and-stick models.

**Figure 10:**
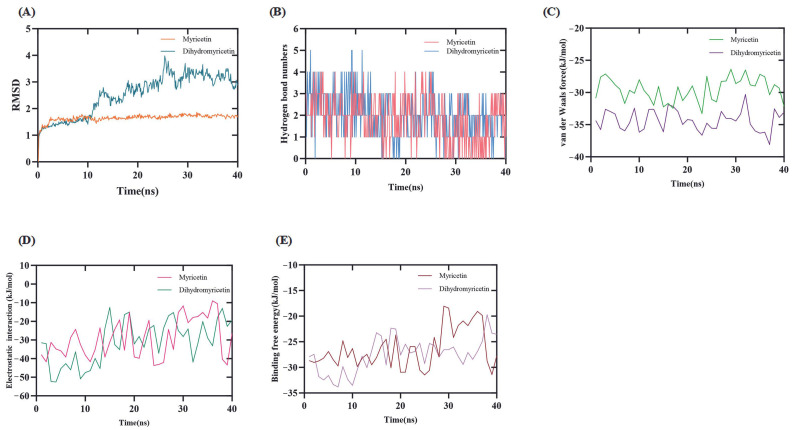
Molecular dynamics analysis of the α-glucosidase complexes with myricetin and dihydromyricetin during the 40 ns simulations. (**A**) Root-mean-square deviation (RMSD) of the protein backbone; (**B**) number of hydrogen bonds formed between α-glucosidase and the flavonoids; (**C**) van der Waals interaction energy; (**D**) electrostatic interaction energy; and (**E**) calculated binding free energy.

Molecular dynamics simulations showed that both myricetin and dihydromyricetin remained associated with α-glucosidase during the 40 ns simulation, as shown in Figure 8. However, clear differences were observed between the two complexes. The RMSD of the α-glucosidase–myricetin complex reached equilibrium rapidly and remained relatively stable, whereas the α-glucosidase–dihydromyricetin complex exhibited larger fluctuations during the later stage of the simulation [69]. This result indicated that myricetin formed a more conformationally stable complex with α-glucosidase than dihydromyricetin. Both flavonoids formed dynamic hydrogen bonds with α-glucosidase throughout the simulation, although the number of hydrogen bonds fluctuated over time. Dihydromyricetin generally showed more favorable van der Waals interaction energy, while the electrostatic interaction energies of both complexes fluctuated considerably. The calculated binding free energies also varied during the simulation and showed partial overlap between the two systems [70], as shown in Figure 9. The Figure 10 shows that both myricetin and dihydromyricetin formed relatively stable complexes with α-glucosidase during the simulation. The RMSD analysis showed that both complexes reached equilibrium with relatively limited fluctuations, while the myricetin-bound complex exhibited slightly lower RMSD values, indicating a more stable conformational state. Moreover, the interaction energy analysis suggested that myricetin maintained stronger ligand–protein interactions than dihydromyricetin, which may be attributed to the presence of the C2=C3 double bond that contributes to a more rigid molecular conformation and facilitates stable interactions with α-glucosidase. Overall, myricetin exhibited greater structural stability within the α-glucosidase complex, whereas dihydromyricetin showed stronger van der Waals contributions but greater conformational fluctuations. These findings suggest that the C2=C3 double bond of myricetin may help maintain a more stable binding conformation with α-glucosidase.

### 3.9. Isothermal Titration Calorimetry Analysis

**Figure 11:**
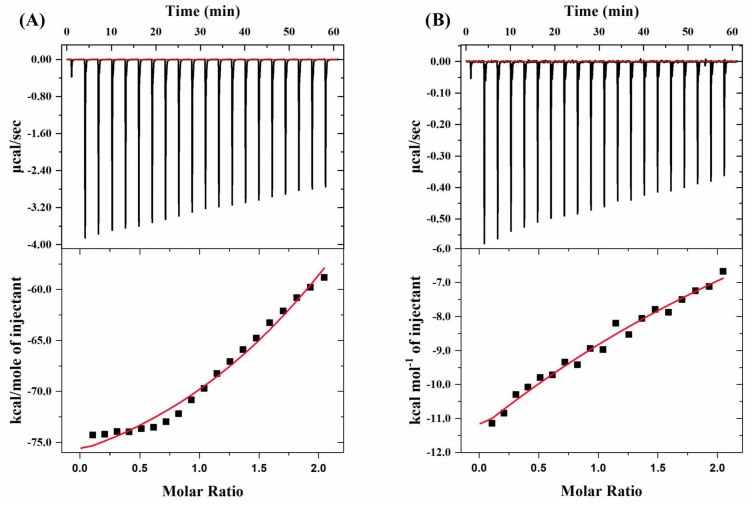
ITC thermograms for the titration of α-glucosidase with two flavonoids. (**A**) Myricetin, and (**B**) Dihydromyricetin.

As almost all binding interactions generate or consume heat, ITC is performed under constant temperature conditions to record the resulting thermal changes, from which the binding affinity (K_D_) can be analyzed thermodynamically [44]. In recent years, it has been successfully applied to an increasing number of enzyme inhibitor complex studies [24]. In this study, ITC was employed to determine the binding affinity (K_D_) of two flavonoids to α-glucosidase (Figure 11). Myricetin and dihydromyricetin both exhibited reversible, non-covalent binding with α-glucosidase, but with markedly different binding affinities. Each heat peak in Figure 11 demonstrated a downward negative peak, representing the release of heat with each injection. The integrated heat of each injection was fitted to the molar ratio of the inhibitor to α-glucosidase through the one-site binding model, and the thermodynamic parameters were obtained. The K_D_ of myricetin was 6.215 ± 0.022 μM, much lower than that of dihydromyricetin (232.648 ± 1.236 μM), indicating a stronger interaction between myricetin and α-glucosidase.

Thermodynamic analysis showed that the interactions of both flavonoids with α-glucosidase were primarily driven by enthalpic contributions and accompanied by increased molecular ordering (Table 2). This behavior suggested that hydrogen bonding played an important role in the formation of the enzyme–inhibitor complexes, in agreement with the molecular docking results. Collectively, the thermodynamic parameters characterized the energetic changes associated with binding and provided further insight into the dominant interaction forces and relative affinities of myricetin and dihydromyricetin for α-glucosidase.

**Table 2:** Thermodynamic parameters for the binding of two flavonols with α-glucosidase measured by ITC.

Samples	ΔH (kJ/mol)	ΔS (J/mol/deg)	ΔG (kJ/mol)	K_D_ (μM)
Myricetin	−343.30 ± 7.31 ^a^	−1050.18 ± 54.39 ^a^	−30.34 ± 8.30 ^b^	6.17 ± 1.14 ^a^
Dihydromyricetin	−17.99 ± 3.56 ^b^	−589.94 ± 20.92 ^b^	−167.49 ± 9.68 ^a^	232.55 ± 12.43 ^b^

Note: Different lowercase letters indicate significant differences among groups (*p* < 0.05).

### 3.10. Surface Plasmon Resonance Analysis

**Figure 12:**
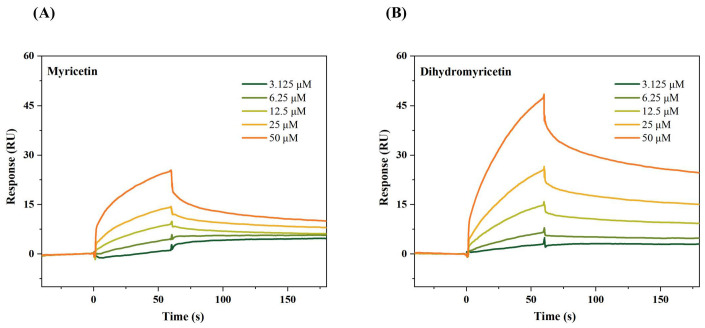
SPR plots of two flavonoids bound to α-glucosidase. (**A**) Myricetin and (**B**) dihydromyricetin. c(myricetin) = c(dihydromyricetin) = 3.125, 6.25, 12.5, 25, 50 μM.

It is worth noting that the binding process of myricetin and dihydromyricetin to α-glucosidase was elucidated for the first time. Based on the evaluation software of the Biacore T200, the binding graphs of myricetin and dihydromyricetin with α-glucosidase and the corresponding parameters were obtained and displayed in Figure 12 and Table 3, respectively. Within the concentration range of 3.125–50 μM, the binding and dissociation of myricetin and dihydromyricetin to α-glucosidase were analyzed. The equilibrium dissociation constant K_D_ is a key indicator of the strength of molecular binding, reflecting the tightness of interaction between the ligands (myricetin and dihydromyricetin) and the target protein (α-glucosidase) [71]. Specifically, K_D_ is the ratio of the dissociation rate constant (K_D_) to the association rate constant (K_a_), calculated by the formula K_D_ = K_d_/K_a_ [72]. A lower K_D_ value indicates a more stable binding of the ligand to the protein, that is, a stronger affinity; whereas a higher K_D_ value suggests a looser binding, weaker affinity, and a propensity for dissociation [51]. K_D_ is of great significance for understanding how drug molecules interact with biological targets and is widely used in biochemical, pharmacological, and molecular biological research [23]. The results indicated that myricetin (K_D_ = 3.416 ± 0.015 μM) had a significantly stronger binding affinity for α-glucosidase than dihydromyricetin (K_d_ = 11.539 ± 0.056 μM), which was consistent with the outcomes of the ITC experiment. This was likely due to the presence of a C2=C3 double bond in myricetin, whereas dihydromyricetin had a saturated single bond at the corresponding position [73]. This structural difference may have affected their binding modes to α-glucosidase [50]. Saturation of the C2=C3 double bond may alter the conjugation, molecular planarity, and three-dimensional conformation of dihydromyricetin, thereby affecting its recognition and binding mode toward α-glucosidase. Simultaneously, the α-glucosidase active site probably has specific polarity requirements, and the polarity of myricetin might be better suited for such binding [55].

Further analysis of the binding process revealed that myricetin (k_a_ = 1668 ± 23 M^−1^ s^−1^) bound to α-glucosidase more rapidly than dihydromyricetin (k_a_ = 339.7 ± 17.1 M^−1^ s^−1^) (Table 3). Myricetin, by occupying the drug’s target site more swiftly, could effectively avoid interference from other competitive molecules, which could contribute to enhancing the drug’s specificity and therapeutic efficacy. Similarly, myricetin (k_d_ = 5.605 ± 0.091 s^−1^) dissociated from α-glucosidase at a higher rate, while dihydromyricetin (k_d_ = 3.921 ± 0.114 s^−1^) exhibited a relatively slower dissociation rate, indicating that its binding to α-glucosidase was more stable. The saturation of the double bond in dihydromyricetin might result in different stereochemical properties, which could also affect its biological activity and pharmacokinetic characteristics [74]. Molecular docking results had potentially explained this, as a salt bridge had formed between dihydromyricetin and the Arg176 residue of α-glucosidase, thereby enhancing the stability of their binding. The overall results of the SPR experiment are consistent with the results of the previous experiments. It has been reported that the double bond on the C ring could enhance binding to the enzyme’s active site, which explains the higher binding affinity of myricetin for α-glucosidase [62,75,76]. While ITC gives a thermodynamic profile, SPR focuses on the kinetics of the interaction. Combining both techniques allows researchers to fully elucidate how AGIs bind to enzymes, revealing both the strength and speed of the interactions, thereby enhancing the understanding of the inhibition mechanism.

## 4. Discussion

Despite their similar flavonoid skeletons, myricetin and dihydromyricetin showed markedly different inhibitory effects on α-glucosidase [52]. The IC_50_ of myricetin was 13.648 ± 0.157 μM, more than 30-fold lower than that of dihydromyricetin, while its K_i_ was also substantially lower [77]. These results demonstrate that saturation of the C2=C3 double bond strongly affects the α-glucosidase-inhibitory activity of these compounds. However, myricetin remained less potent than acarbose, indicating that its main significance lies in revealing the structure–activity relationship of flavonoid inhibitors rather than replacing established inhibitors directly.

The kinetic results further showed that myricetin acted as a competitive inhibitor, whereas dihydromyricetin exhibited non-competitive inhibition [78]. The C2=C3 double bond contributes to the conjugation and planarity of myricetin, which may facilitate its entry into the catalytic pocket and allow it to compete directly with the substrate. Saturation of this bond changes the three-dimensional conformation of dihydromyricetin and may redirect its interaction toward an allosteric region. Thus, the structural difference between the two flavonoids affected not only inhibitory potency but also the mode of enzyme recognition.

The fluorescence results supported their different interaction behaviors [79]. Both flavonoids caused concentration-dependent quenching of α-glucosidase fluorescence, but myricetin produced a greater decrease than dihydromyricetin. Synchronous fluorescence indicated that myricetin mainly affected the microenvironment surrounding Trp residues, whereas dihydromyricetin exerted a greater influence on Tyr residues [80]. Three-dimensional fluorescence also showed that myricetin caused a larger decrease in aromatic-residue-related fluorescence. These results suggest that myricetin interacts more strongly with α-glucosidase and produces a more pronounced perturbation of its local microenvironment. Nevertheless, these fluorescence changes should be interpreted as conformational perturbations rather than direct evidence of extensive protein unfolding.

The different inhibitory activities of myricetin and dihydromyricetin against α-glucosidase were mainly associated with their structural differences, particularly the saturation state of the C2=C3 double bond in the C-ring. The presence of the C2=C3 double bond in myricetin contributes to a more planar and rigid molecular conformation, which may facilitate a better spatial arrangement of hydroxyl groups and enhance interactions with the enzyme catalytic site through hydrogen bonding and hydrophobic interactions, thereby favoring a stable competitive binding mode [81]. In contrast, hydrogenation of this double bond in dihydromyricetin increases molecular flexibility and alters the orientation of functional groups, potentially reducing its ability to achieve optimal spatial complementarity with the catalytic region and weakening its interaction with α-glucosidase. Consequently, dihydromyricetin may preferentially interact with alternative binding regions through non-catalytic site interactions, resulting in a noncompetitive inhibition pattern. Molecular docking and molecular dynamics simulations further supported this structure–activity relationship by revealing differences in the binding modes and conformational stability of the two compounds [82]. Specifically, the rigid myricetin–enzyme complex rapidly equilibrated and remained conformationally stable over the 40 ns trajectory, whereas the inherently flexible dihy-dromyricetin led to a fluctuating complex with larger RMSD variations in the later stages. These in silico findings provided theoretical support for the distinct inhibitory behaviors of myricetin and dihydromyricetin, while further validating the influence of C-ring structural features on their interactions with α-glucosidase.

The ITC and SPR results experimentally confirmed the stronger binding of myricetin. ITC showed that the K_D_ of myricetin was approximately 6.215 ± 0.022 μM, compared with approximately 232.648 ± 1.236 μM for dihydromyricetin. Both interactions were enthalpically favorable, supporting an important contribution from hydrogen bonding and other specific non-covalent interactions [82]. SPR similarly showed a lower K_D_ for myricetin and revealed that its association rate was approximately five times higher than that of dihydromyricetin. Although myricetin dissociated slightly faster, its much faster association resulted in stronger overall affinity [83]. The differences between the absolute ITC and SPR K_D_ values may result from differences in temperature, buffer conditions, protein immobilization, and fitting methods, but both techniques consistently identified myricetin as the stronger ligand.

Overall, the results support the interpretation that the C2=C3 double bond contributes to the planarity, catalytic-site recognition, binding stability, and inhibitory potency of myricetin. Saturation of this bond in dihydromyricetin alters its binding orientation and favors a weaker, non-competitive interaction. The agreement among enzyme kinetics, fluorescence spectroscopy, molecular docking, MD simulation, ITC, and SPR strengthens this structure–activity relationship. However, the study used yeast α-glucosidase and a homology-based structural model; therefore, further validation using mammalian or recombinant human intestinal α-glucosidases and longer independent MD simulations is required [84].

## 5. Conclusions

This study systematically compared the inhibitory mechanisms of myricetin and dihydromyricetin against α-glucosidase using enzyme kinetics, multispectral analysis, molecular docking, isothermal titration calorimetry and surface plasmon resonance. In summary, compared to dihydromyricetin, myricetin was found to competitively bind to the active site of α-glucosidase primarily through hydrogen bonding and hydrophobic interactions, leading to a more pronounced conformational change in the enzyme. Furthermore, it exhibited a faster binding rate and higher affinity, resulting in stronger inhibitory activity against α-glucosidase. These findings collectively demonstrate that the C2=C3 double bond plays a crucial role in enhancing the interaction between flavonoids and α-glucosidase, thereby improving inhibitory activity. This work provides new insights into the structure–activity relationship of flavonoid-based α-glucosidase inhibitors and offers a useful experimental framework for investigating enzyme–inhibitor interactions, supporting the development of natural antidiabetic agents and functional food ingredients.

## Figures and Tables

**Table 1:** Docking score energies and interaction residues of two flavonoids with α-glucosidase.

Comp.	Binding Energy (kcal/mol)	Binding Site	Hydrogen Bond Interactions	Hydrophobic Interactions	Other Interactions
Myricetin	−8.9	catalytic	Lys13, Ile272×2	Trp15, Ala292	
Dihydromyricetin	−7.8	allosteric	Ser162, Arg 176×2	Asn417	Arg176 (Salt bridges)
p-NPG ^a^	−7.5	catalytic	Ile262, Arg263×2, Val266, Ile272×2	Asn259×2, Ile272, Thr274, His295×2	

^a^ Substrate for α-glucosidase.

**Table 3:** Binding constants of two flavonoids with α-glucosidase.

Samples	k_a_ (M^−1^ s^−1^)	k_d_ × 10^−3^ (s^−1^)	K_D_ (μM)
Myricetin	1668.153 ± 23.247 ^b^	5.605 ± 0.091 ^b^	3.361 ± 0.834 ^a^
Dihydromyricetin	339.724 ± 17.041 ^a^	3.921 ± 0.112 ^a^	11.542 ± 1.591 ^b^

Note: Different lowercase letters indicate significant differences among groups (*p* < 0.05).

## Data Availability

The original contributions presented in the study are included in the article. Further inquiries can be directed to the corresponding authors.
